# Analysis of Cumulative Cancer Risk Associated with Disinfection Byproducts in United States Drinking Water

**DOI:** 10.3390/ijerph17062149

**Published:** 2020-03-24

**Authors:** Sydney Evans, Chris Campbell, Olga V. Naidenko

**Affiliations:** Environmental Working Group, 1436 U Street NW, Suite 100, Washington, DC 20009, USA; chris@ewg.org (C.C.); olga@ewg.org (O.V.N.)

**Keywords:** drinking water, disinfection byproducts, cancer risk, bladder cancer, cumulative risk assessment

## Abstract

Hundreds of different disinfection byproducts form in drinking water following necessary treatment with chlorine and other disinfectants, and many of those byproducts can damage DNA and increase the risk of cancer. This study offers the first side-by-side comparison of cancer risk assessments based on toxicological and epidemiological studies of disinfection byproducts using a comprehensive contaminant occurrence dataset for haloacetic acids and trihalomethanes, two groups of disinfection byproducts that are regulated in drinking water. We also provide the first analysis of a new occurrence dataset for unregulated haloacetic acids that became available from the latest, fourth round of the U.S. EPA-mandated unregulated contaminant monitoring program (UCMR4). A toxicological assessment indicated that haloacetic acids, and in particular brominated haloacetic acids, are more carcinogenic and are associated with a greater number of attributable cancer cases than trihalomethanes. Based on the toxicological analysis, cumulative lifetime cancer risk due to exposure to trihalomethanes and haloacetic acids for community water systems monitored under UCMR4, estimated with standard default parameters for body weight and water intake, corresponds to 7.0 × 10^−5^ (3.5 × 10^−5^–1.3 × 10^−4^). The same analysis conducted with age sensitivity factors to account for elevated risk in infants and children yielded a cumulative risk estimate of 2.9 × 10^−4^ (1.7 × 10^−4^–6.2 × 10^−4^). Epidemiological data suggest that lifetime cancer risk from disinfection byproducts for the U.S. population served by community water systems is approximately 3.0 × 10^−3^ (2.1 × 10^−4^–5.7 × 10^−3^), or a lifetime cancer risk of three cases per thousand people. Overall, this analysis highlights the value of using human data in health risk assessments to the greatest extent possible.

## 1. Introduction

Drinking water treatment with disinfectants, such as chlorine, chloramine, and ozone creates a variety of reactive chemical intermediates and disinfection byproducts that may be harmful to human health and the environment [1,2]. Chlorine-based water disinfection, introduced at the beginning of the 20th century, has saved countless lives and drastically decreased the incidence of water-borne microbial diseases transmitted through drinking water. However, epidemiological research has reported associations between adverse health effects and the presence of disinfection byproducts in tap water at concentrations that generally meet applicable national drinking water standards [3]. Multiple studies have reported an increased risk of bladder cancer in association with exposure to drinking water disinfection byproducts [4,5,6], and the risk of other cancers has been suggested [7,8]. Birth defects [9] and miscarriages [10] have also been linked to disinfection byproducts. Toxicity and carcinogenicity of disinfection byproducts are mediated through pathways involving genotoxicity, cell cycle disruption, and oxidative stress [11].

The majority of epidemiological studies of water disinfection byproducts have focused on the combined exposure to four trihalomethanes (THM4): chloroform, bromoform, dibromochloromethane, and bromodichloromethane. The THM4 group, defined by the U.S. Environmental Protection Agency (EPA) as “total trihalomethanes” or TTHM, is regulated with a single maximum contaminant level of 80 µg/L in drinking water. This legal limit was established based on the costs and benefits of various water disinfection alternatives [12]. In addition to the THM4 group, the U.S. EPA also set maximum contaminant levels for several other disinfection byproducts: bromate, chlorite, and a group of five haloacetic acids (HAA5). The HAA5 group includes monochloroacetic acid, dichloroacetic acid, monobromoacetic acid, dibromoacetic acid, and trichloracetic acid; the entire group is regulated with a single maximum contaminant level of 60 µg/L in drinking water. The concentration and composition of disinfection byproduct mixtures in drinking water are influenced by a number of factors, such as the presence of natural organic matter in the supply water, pH, temperature, additional halogens such as bromide and iodide, and other co-occurring contaminants or water treatment chemicals [13]. A wide variety of halogenated and nitrogenated substances can form during water disinfection, and one recent review estimated that over a thousand different chemical contaminant species may occur [14]. Exposure data are primarily available for the regulated disinfection byproducts, particularly the THM4 and HAA5 groups, since community water systems are required to monitor the levels of these contaminants and report the data to their state drinking water authorities. However, human health risks reported in epidemiological studies are likely due to mixtures of disinfection byproducts, marked by the presence of THMs and HAAs in drinking water. 

Given that water disinfection is essential for protecting public health from waterborne microbial diseases, a better understanding of the risks of exposure to disinfection byproducts, as well as ways to minimize that risk, is needed. Here we present an assessment of cumulative cancer risk due to disinfection byproducts based on toxicological and epidemiological data. The approach outlined in our study offers a valuable opportunity to compare human and animal data and to consider how such data can be used in risk assessments.

## 2. Materials and Methods

### 2.1. Exposure Assessment for Trihalomethanes and Haloacetic Acids in Drinking Water

This study utilizes two sets of disinfection byproduct occurrence data: the U.S. EPA’s Unregulated Contaminant Monitoring Rule 4 (UCMR4) dataset and occurrence data for trihalomethanes and haloacetic acids for the years 2014–2017, available in a comprehensive contaminant occurrence dataset for over 48 thousand community water systems in the U.S. [15], described previously [16,17,18,19]. The UCMR4 monitoring spans over three years, from 2018 to 2020, and data are posted on the EPA website as available prior to the official completion of the program. In this analysis, we used the latest accessible UCMR4 data that were posted in January 2020 [20], including data for 3579 community water systems serving a combined population of approximately 183 million people. The UCMR4 program has not yet been officially completed. Thus, the HAA occurrence information may slightly change for the final completed dataset, whenever it becomes publicly available. Nevertheless, even at this stage, the dataset offers extensive and valuable information on the occurrence of haloacetic acid groups in drinking water in the U.S.

This analysis includes data for four regulated trihalomethanes (THM4, including chloroform, bromoform, bromodichloromethane, and dibromochloromethane) and for nine haloacetic acids listed in Table 1. The UCMR4 dataset does not include concentration data for individual compounds and only reports group concentrations for the regulated HAA5 group, the HAA6Br group of six brominated haloacetic acids, and HAA9, which includes all nine chlorinated and brominated haloacetic acids (Table 2). All contaminant concentration values are reported in micrograms per liter (µg/L). 

Population statistics for community water systems were obtained from state drinking water programs and supplemented with information from the U.S. EPA Envirofacts database [21]. Classifications of systems as groundwater or surface water follow the EPA’s Safe Drinking Water Information System (SDWIS) classifications. The total population served by community water systems per state was validated with 2017 census state-level population estimates available from U.S. Census data [22] and the estimated population served by private water sources, available from the U.S. Geological Survey [23]. For states where population estimates for the number of people served by community water systems differed from the U.S. Census data for the state, we applied a state-specific adjustment factor described in a prior publication [19]. All population numbers represent an estimate, and the specific number of customers served by community water systems may differ.

For contaminant concentrations, we calculated population-weighted averages (C_PW_) based on average contaminant concentrations in community water systems, according the formula:

(1) $$C_{\mathrm{PW}}=\Sigma \left[C_{\mathrm{system}}\times \left(\frac{P_{\mathrm{system}}}{P_{\mathrm{total}}}\right)\right]$$

where the average contaminant concentration (C_system_) and system population (P_system_) refer to any individual water system included in the data set, total population (P_total_) corresponds to the total population served by all community water systems in the dataset, and these factors are summed for all community water systems included in the dataset. Test data points for contaminants reported as “non-detect” are assigned the value of zero and included in all calculations in this study. Since it is possible that contaminants may be present at levels above zero but below the official limit of detection for reporting purposes, this approach for calculating averages is conservative and the calculated average values may be lower than the true national population-weighted averages.

Among the haloacetic acids, the national contaminant occurrence dataset is more robust for the constituents of the HAA5 group and is limited for four unregulated brominated haloacetic acids (Table 1) due to lack of nationwide monitoring requirements for those chemicals. Analysis for tribromoacetic acid, bromochloroacetic acid, bromodichloroacetic acid and chlorodibromoacetic acid relies on data from a smaller number of water systems, ranging between approximately one to eight thousand systems. For this study, we are making an assumption that the relative presence of individual disinfection byproducts in the available dataset is comparable to the concentrations for regulated contaminants on the national level. This assumption represents an uncertainty that will need to be addressed by future research. 

### 2.2. Calculation of Lifetime Cancer Risk Based on Toxicological Studies of Disinfection Byproducts

Human-equivalent cancer slope factors calculated by the authors were derived using the U.S. EPA Benchmark Dose Modeling Software (BMDS), version 2.7 [24]. Datasets from National Toxicology Program animal studies on dibromoacetic acid, bromochloroacetic acid, and bromodichloroacetic acid were chosen for modeling based on the quality, suitability, and sensitivity of the studies, as well as the range of reported cancer sites and endpoints [25,26,27]. The two-year studies in rats and mice were chosen for modeling to reduce uncertainties regarding dosing and duration adjustments. Animal cancer incidence data deemed suitable using standard protocols and recommendations from benchmark dose modeling technical guidance documents were analyzed using the multi-stage cancer model [28,29].

After examining and modeling a number of endpoints with suitable animal dose-response data, combined response data for hepatocellular adenomas, hepatocellular carcinomas, and hepatoblastomas for male mice (dibromoacetic acid, bromochloroacetic acid) and female mice (bromodichloroacetic acid) were chosen as the most sensitive endpoint for determining a point of departure or benchmark dose [25,26,27]. These values were used to calculate the human-equivalent cancer slope factors after applying standard bodyweight transformations [30].

For toxicologically-estimated cancer risk based on animal studies, contaminant concentrations associated with a specific cancer risk level can be calculated from the cancer slope factor, taking into consideration daily water intake, body weight, and age susceptibility. This calculation, based on the U.S. EPA methodology [31], is

(2) $$\mathrm{CSF}=\frac{\mathrm{BMR}}{\mathrm{POD}}$$

where benchmark response (BMR) can be an excess risk of 5%, 10%, or a different number used for benchmark dose modeling [28]. The point of departure (POD) or benchmark dose is derived from benchmark dose modeling of tumor incidence data from animal bioassays, and can represent either the central estimate of the benchmark dose or the upper or lower 95% confidence limit. Cancer slope factors can be converted to the human equivalent following

(3) $${\mathrm{CSF}}_{\mathrm{human}}={\mathrm{CSF}}_{\mathrm{animal}}\times {\left(\frac{{\mathrm{BW}}_{\mathrm{human}}}{{\mathrm{BW}}_{\mathrm{animal}}}\right)}^{\frac{1}{4}}$$

where BW_human_ is the default weight for human adults, 70 kg, and BW_animal_ corresponds to the average weight of animals in the control group of the animal bioassay used for the cancer slope determination. The formula is adapted from that used by the California Office of Environmental Health Hazard Assessment (OEHHA) [30].

The cancer risk-specific benchmark concentration (B) is

(4) $$B=\frac{R}{{\mathrm{CSF}}_{\mathrm{human}}\times \left(\frac{\mathrm{DWI}}{\mathrm{BW}}\right)}$$

where the risk level (R) can be one-in-a-million (10^−6^) or a different value. The cancer slope factor (CSF_human_) is expressed in (mg/kg-day)^−1^, average water intake (DWI) is L/day, and body weight (BW) is in kg. The interpretation of this calculation is that the daily ingestion of drinking water with this specified contaminant level over the course of a lifetime would increase lifetime cancer risk by the specific risk levels, such as one-in-a-million. Throughout this article, cancer risk-specific benchmarks are expressed in the units of µg/L.

For calculating cancer risk-specific benchmarks, water intake and body weight parameters are derived from national statistics information. EPA’s standard approach has used the default values for daily water intake of 2 L and adult body weight of 70 kg. However, body weight and water intake differ by age and gender [32]. For 2015–2016, U.S. National Health Statistics Reports calculated mean body weight in the American population at 89.8 kg for men and 77.4 kg for women [33]. In a 2004 analysis, the U.S. EPA reported the mean for direct and indirect community water ingestion for all ages and genders at 0.926 L (with a 90% interval of 0.903–0.949) and the 95th percentile at 2.54 (2.50–2.58) L/day [34]. The 95th percentile corresponds to 0.043 (0.41–0.44) L/kg daily water intake [34]. OEHHA used the upper 95th percentile of consumption of municipal water for the general population of 0.044 L/kg for establishing a cancer-risk specific benchmark for bromate [35]. Additionally, consumption of tap water changes over time, with trends for greater consumption of bottled water and more common use of in-home water filtration devices [36]. Concerns about tap water safety and discoveries of tap water contamination decrease municipal water consumption as residents in the affected communities turn to bottled water [36].

To account for heightened susceptibility of the fetus, infant, and developing child to chemical toxicity [37,38,39,40], age sensitivity factors are incorporated into the formula for deriving health-protective contaminant concentrations. The state of Minnesota Department of Health has published the following age-dependent susceptibility factors for calculation of 70-year lifetime cancer unit risk: a factor of 10 for age 0–2 (infant) and a factor of 3 for age 2–16 (child) [38]. California OEHHA used the same age sensitivity factors and also added the age-sensitivity factor of 10 for the exposure of the fetus during the 3rd trimester [41]. Incorporation of these factors into toxicological risk assessment for cancer is based on established research demonstrating greater cancer risk associated with exposure to carcinogenic substances at a younger age [38,39,40]. Here we followed California OEHHA’s estimates for the upper 95th percentile drinking water intake and age-sensitivity factor adjustments for trihalomethanes [42] and haloacetic acids [30], with adjusted drinking water consumption intakes of 0.18 and 0.129 L/kg-day, respectively.

Benchmark cancer risk concentrations for a group of contaminants (B_group_) are calculated following the method presented in an analysis by California OEHHA in 2010 for the group of trihalomethanes [37] and described in an earlier study [16] as

(5) $$B_{\mathrm{group}}=\Sigma \left(C_{\mathrm{PW}}\right)÷\Sigma \left(\frac{C_{\mathrm{PW}}}{B}\right)$$

where contaminant concentration averages (C_PW_) are national, population-weighted averages as listed in Table 1, and sums are for all contaminants included in a group. 

Lifetime cancer risk due to the presence of a contaminant is calculated as

(6) $$R=\left(\frac{C}{B}\right)\times 10^{-6}$$

Cancer risks (R) for individual contaminants are treated in a response additive manner, following the approach that U.S. EPA uses for cumulative cancer risk assessment of air pollutants [39,40]. With this simple additive approach, national lifetime cancer risk (R_national_) was calculated as

(7) $$R_{\mathrm{national}}=\frac{\Sigma \left(R_{\mathrm{system}}\times P_{\mathrm{system}}\right)}{P_{\mathrm{total}}}$$

### 2.3. Calculation of Lifetime Cancer Risk Based on Epidemiological Studies of Disinfection Byproducts

Calculations for bladder cancer risk and attributable case counts (A_cases_) followed previously published methodology [19], according to the formula

(8) $$A_{\mathrm{cases}}=\left[\Sigma \left(P_{\mathrm{exposed}}\times \Delta \mathrm{OR}\right)\right]\times I_{\mathrm{baseline}}$$

where each exposed population (P_exposed_) is the number of people exposed to drinking water with THM4 concentrations within a specified concentration range; ∆OR is the difference in odds ratios or risk ratios between the specified exposed population and the unexposed population; and baseline incidence (I_baseline_) is the estimated proportion of the cumulative cancer incidence that would be present in the absence of exposure. 

Odds ratios for increased bladder cancer risk in relation to THM4 concentrations come from the 2015 publication by Regli and colleagues [6], which specifies both the central estimate and the upper and lower confidence limits for cancer risk observed in epidemiological studies in 10 µg/L intervals. For this study, odds ratios and relative risk ratios are considered interchangeable since cancer is a rare event [43]. Odds or risk ratios for the unexposed population are assumed to be 1.0 for this calculation, reflecting the expected absence of excess risk when there is no exposure to disinfection byproducts in drinking water.

Baseline incidence is estimated as a proportion of cumulative incidence and is calculated as

(9) $$I_{\mathrm{baseline}}=\frac{I_{\mathrm{cumulative}}\times P_{\mathrm{total}}}{\left(\Sigma \left(P_{\mathrm{exposed}}\times \mathrm{OR}\right)\right)+\left(P_{\mathrm{unexposed}}\right)}$$

where the cumulative incidence (I_cumulative_) is the lifetime risk of cancer diagnosis in the total population, the unexposed population (P_unexposed_) is the total population less the exposed populations and other values, as described previously.

The cancer risk attributable to exposure to disinfection byproducts (A_risk_) can then be calculated as

(10) $$A_{\mathrm{risk}}=\frac{A_{\mathrm{cases}}}{P_{\mathrm{total}}}$$

where the total population (P_total_) is the total population served by community water systems.

This analysis uses the national lifetime risk of diagnosis of bladder cancer, calculated with the DevCan software [44] (https://surveillance.cancer.gov/devcan/). The possibility of developing bladder cancer in a lifetime of 95 years is calculated using the cross-sectional counts of incident cases in males and females between 2014 and 2016 as reported through the NCI Surveillance, Epidemiology, and End Results (SEER) program [45].

### 2.4. Calculation of Economic Costs due to Estimated Bladder Cancer Cases Associated with Disinfection Byproducts

The economic cost of cancer cases was calculated as the direct medical cost in 2015 U.S. dollars. Annualized mean net costs of care per patient for bladder cancer published by the National Cancer Institute were based on research by Mariotto and colleagues [46]. Cost per case was calculated as

(11) $$E_{\mathrm{case}}=E_{\mathrm{initial}}+E_{\mathrm{continuing}}+E_{\mathrm{final}}$$

where the continuing cost (E_continuing_) was multiplied by median years lived with disease less two for the first and last year. Median years lived with disease was calculated as the difference between median age at death and median age at diagnosis as reported by National Cancer Institute, and the average of costs for men and women was included here. Cost for last year of life (E_final_) was the average of the two values reported as cancer death and other cause of death. Values were converted from 2010 U.S. dollars to 2015 U.S. dollars by using the Bureau of Economic Analysis Personal Consumption Expenditures Health Index. To address volatility in the health care index, the blended account index was used, as recommended by Bureau of Economic Analysis [47]. 

Values reported in the text have been rounded to two or three significant digits. Due to rounding, some totals in the tables may not correspond precisely to sums of the reported values.

## 3. Results

### 3.1. Exposure Assessment for Trihalomethanes and Haloacetic Acids in Drinking Water

**Figure 1:**
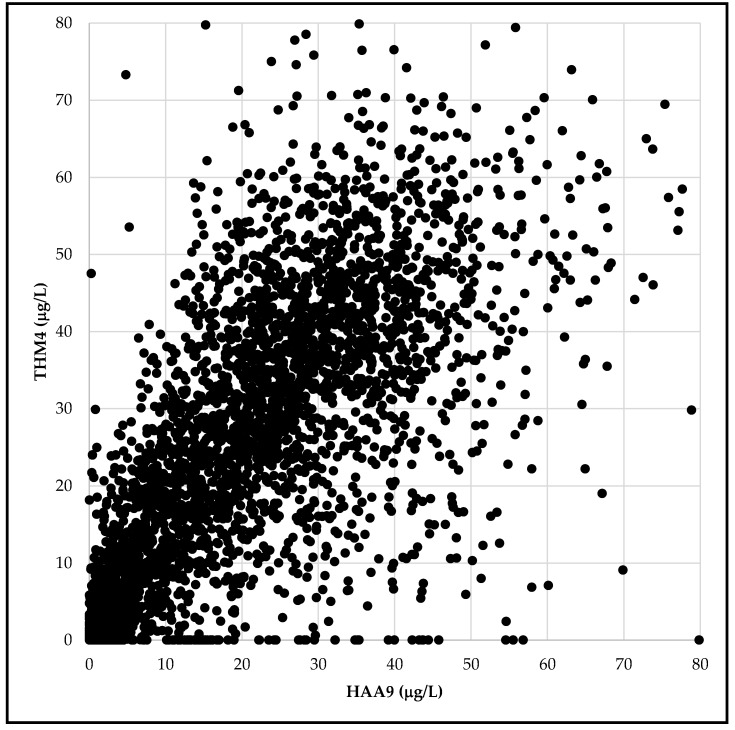
The average concentration of trihalomethanes (THM4) in community drinking water systems according to compliance testing results for 2014 to 2017, and the average concentration of haloacetic acids (HAA9) as reported in the EPA Unregulated Contaminant Monitoring Rule 4 occurrence dataset for testing conducted in 2018 to 2019 for 3579 community water systems.

Based on UCMR4 data, we calculated population-weighted averages of 24.9 µg/L for HAA9, 19.1 µg/L for HAA5, and 7.0 µg/L for HAA6Br for the systems included in UCMR monitoring (Table 2). In an earlier study, we reported the population-weighted average of THM4 in the United States community water supplies as 26 µg/L [16]. While relative concentrations of individual disinfection byproducts depend on multiple water chemistry conditions, there is an overall correlation between trihalomethanes and haloacetic acids, as anticipated (Figure 1).

**Figure 2:**
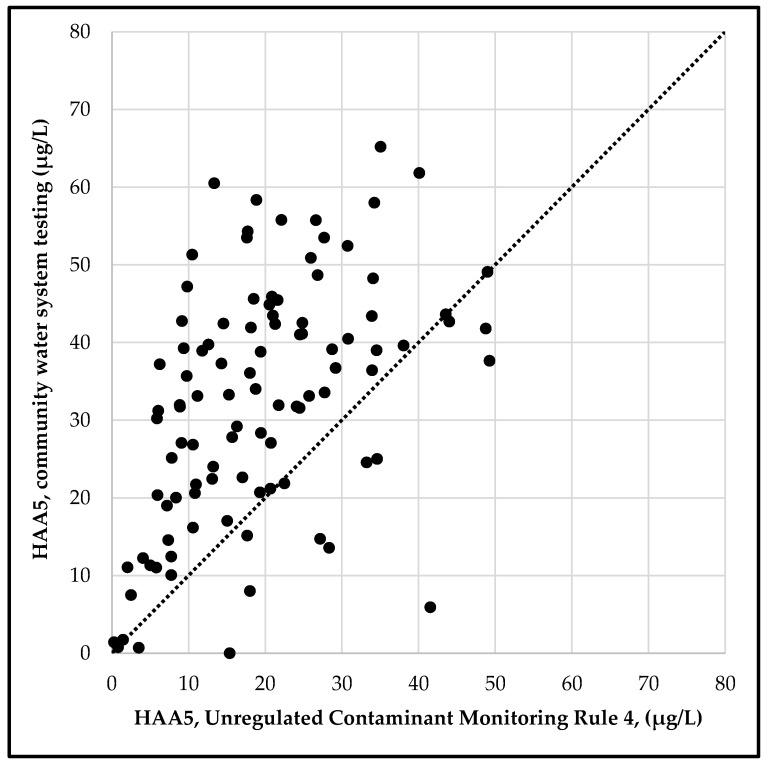
The average concentration of regulated haloacetic acids (HAA5) in community drinking water systems as reported by utilities for 2014 to 2017, and the average concentration of regulated haloacetic acids (HAA5) as reported in the EPA Unregulated Contaminant Monitoring Rule 4 occurrence dataset for testing from 2018 to 2019 for the 100 largest community water systems, which serve a combined population of 74 million. The dotted line indicates where values from both datasets would be identical.

The UCMR-monitored systems serve an estimated 183 million people, approximately 65% of the population served by community water systems in the U.S. We compared average HAA5 levels reported in the UCMR4 dataset with average HAA5 levels for the same utilities in the 2014–2017 period based on compliance testing data in the comprehensive dataset. We selected the hundred largest water systems, serving a combined population of over 74 million. As Figure 2 demonstrates, there is a clear correlation of the reported HAA5 concentrations for each dataset, indicating consistency between the two sources, yet the values are not identical. Further, the levels of disinfection byproducts vary throughout a drinking water distribution system. To address this uncertainty, we adapted the approach of averaging all available sampling values in a given dataset for individual water systems. The resulting average value reflects overall disinfection byproduct concentrations in each system, and concentrations at specific locations in a system may be different.

### 3.2. Calculation of Lifetime Cancer Risk Based on Toxicological Studies of Disinfection Byproducts

Assessment of human cancer risk from animal toxicology data requires a translation of the dose-response data from laboratory animals to humans, accounting for different body sizes and species-specific differences in toxicokinetics and toxicodynamics [48]. The risk calculation can also include factors to account for heightened susceptibility of early life stages, such as the developing fetus, infants, and children [38,41,49]. Following the U.S. EPA methodology, we calculated cancer potency for three brominated disinfection byproducts using Benchmark Dose Modeling Software (Table 3). For this analysis, we used animal bioassay data for dibromoacetic acid, bromochloroacetic acid, and bromodichloroacetic acid from toxicology studies conducted by the National Toxicology Program [25,26,27]. Benchmark responses of 5% or 10% are both used for benchmark modeling in risk assessment [50]. We calculated human-equivalent cancer slope factors based on 95% lower confidence limits of the benchmark doses, following the approach of California OEHHA and the U.S. EPA. Table 3 demonstrates that calculated cancer slope factors are very similar, whether calculated based on 5% or 10% excess risk, suggesting that either approach can be used for assessing cumulative cancer risk for disinfection byproducts. Numerically smaller slope factors indicate weaker carcinogenic potency, while larger slope factors indicate higher carcinogenic potency. According to the available data, bromochloroacetic acid has higher carcinogenic potency compared to dibromoacetic acid and bromodichloroacetic acid (Table 3).

We compiled the reported human cancer slope factors and one-in-a-million cancer risk benchmarks for haloacetic acids and trihalomethanes published by California OEHHA and by the U.S. EPA (Table 4). For calculating cancer slope factors, California OEHHA used an approach with 5% extra risk, while U.S. EPA used 10%, and the two cancer slope values, where available for the same chemical, are overall similar.

Of note, California OEHHA used age-specific water intake and age sensitivity factors for defining cancer risk-specific concentrations, while the U.S. EPA used a default adult body weight of 70 kg, and default daily water intake of 2 L, and did not apply any adjustments for children’s body weight, water intake, or susceptibility to carcinogens. Differences in approaches for calculating risk-specific contaminant concentrations explain why those values are different between California OEHHA and the U.S. EPA, even though the cancer slope factors are broadly similar. The differences between calculated slope factors are likely due to the specific animal bioassay studies chosen for benchmark modeling. Our modeled cancer slope factor for dibromoacetic acid using 5% extra risk is close to the one reported by California OEHHA: a slope factor of 0.210 (mg/kg-day)^−1^ (Table 3) versus a slope factor of 0.250 (mg/kg-day)^−1^ (Table 4 and [30]).

**Figure 3:**
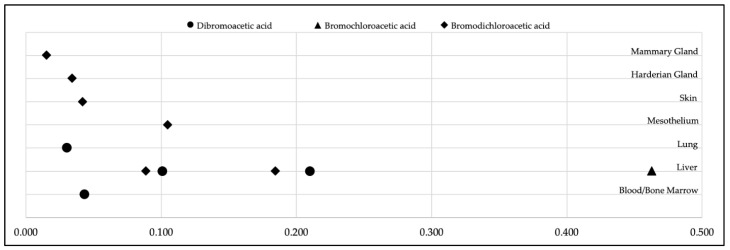
Distribution of human-equivalent cancer slope factors (mg/kg-day^−1^) for dibromoacetic acid, bromochloroacetic acid, and bromodichloroacetic acid, as derived from tumor incidence data [25,26,27] in the tissues of rats and mice using a benchmark response of 5% excess risk and the lower 95% confidence limit on the benchmark dose.

To illustrate the data options associated with the development of cancer slope factors, Figure 3 presents our derivation of human-equivalent cancer slope factors for dibromoacetic acid, bromochloroacetic acid, and bromodichloroacetic acid calculated from tumor incidence in different animal tissues. This comparison highlights how the choice of specific animal bioassay study and modeling approaches influence the calculated cancer slope factors. In National Toxicology Program studies, various types of tumors were observed in laboratory rats and mice exposed to these chemical substances [25,26,27]. Among those tumors and tumor sites, the liver was the most sensitive organ for exposure-related tumor development, as indicated by the highest slope factors. As noted in Table 3, we adapted cancer slope factors for dibromoacetic acid, bromochloroacetic acid and bromodichloroacetic acid based on the liver data. For all subsequent analyses in this study, we used cancer slope factors calculated with a benchmark response of 5% extra risk as the most sensitive approach for the detection of excess cancer risk.

To assess cancer risk due to the presence of haloacetic acids in drinking water, we were faced with a data limitation in the UCMR4 dataset whereby the U.S. EPA reported only the group concentrations for HAA5, HAA6Br, and HAA9 and not the concentrations of individual haloacetic acids. We developed concentration-weighted cancer risk benchmarks for the three HAA groups following Equation (5), as previously described [16,37]. Calculated group risk benchmarks are listed in Table 5 and used for analyses in Table 6.

**Figure 4:**
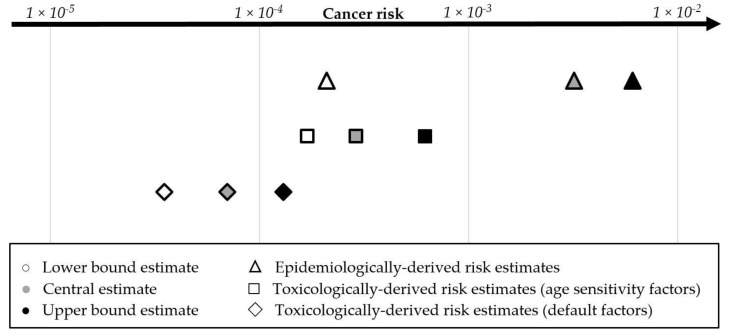
Cumulative assessment of lifetime cancer risk due to disinfection byproducts in drinking water using epidemiologically derived risk estimates based on human studies of disinfection byproducts and bladder cancer and toxicologically derived risk estimates based on animal studies of disinfection byproducts. Values in this figure are calculated for community water systems included in the UCMR4 program.

Cancer bioassay animal studies are not available for chlorodibromoacetic acid and tribromoacetic acid. However, they were both listed by the National Toxicology Program as “reasonably anticipated to be a human carcinogen” based on metabolism data and similarity to other haloacetic acids, which have been tested in animal bioassays [57]. Following a recent study from the scientists at the National Toxicology Program [58], we used a read-across approach for these two haloacetic acids whereby the same cancer risk benchmark is applied to chlorodibromoacetic acid as calculated for bromochloroacetic acid and to tribromoacetic acid as calculated for dibromoacetic acid (Table 5). With the parameters listed, we calculated an additive, concentration-weighted cancer risk benchmark for each group (Table 5). We note that monochloroacetic and monobromoacetic acids are not considered carcinogenic and do not have a cancer risk benchmark. For chloroform, California OEHHA calculated a risk-specific concentration from the geometric mean of cancer slope factors modeled from five different animal bioassays [42]. The benchmark dose from a study published by the U.S. National Cancer Institute [56], with the cancer slope factor closest to this geometric mean, is included in Table 5 and used in later analysis for comparison (Figure 4).

Based on group cancer risk benchmarks and UCMR4 data for the average concentrations of HAA5, HAA6Br, and HAA9 in each water utility in the UCMR program, we calculated the estimated number of lifetime cancer cases attributable to these chemical groups. The upper bound estimate presented in Table 6 represents the number of attributable cases calculated using the lower bound estimate of the benchmark dose, and vice versa for the lower bound estimate of cancer cases. The previously reported value of 47.9 thousand cases for trihalomethanes calculated for the dataset spanning 2010–2017 [16] falls in this calculated range (Table 6). 

For the HAA5 group, we compared the estimates of attributable lifetime cancer cases from a comprehensive national dataset and from the UCMR4 dataset. Calculations for the UCMR4 dataset results in approximately 72% of cases expected for the HAA5 group from the national dataset. This finding makes sense, as the UCMR4 program includes all the large community water systems with a combined population of approximately 183 million people. It is reasonable to hypothesize that the UCMR4 data for HAA6Br and HAA9 underestimate the total nationwide HAA6Br- and HAA9-attributable cases to a similar extent as we report for the HAA5 group. As noted earlier, cancer slope factors and cancer potency are greater for brominated haloacetic acids compared to chlorinated haloacetic acids, and this is reflected in the greater number of attributable cancer cases due to the HAA6Br group relative to the HAA5 group. As expected, the greatest number of attributable cases is calculated for the HAA9 group. Finally, we calculated cumulative cancer risk due to HAA9 and THM4 groups for the subset of systems included in the UCMR4 program. Central estimates for this cumulative lifetime risk range between 7.0 × 10^−5^ and 2.9 × 10^−4^, depending on whether default parameters or age-sensitivity parameters are used to estimate risk (Table 6). 

The read-across approach for chlorodibromoacetic acid and tribromoacetic acids represents an area of uncertainty for our study. Given that trichloroacetic acid is a more potent carcinogen compared to dichloroacetic acid, we hypothesize that a similar cancer potency relationship would apply for dibromoacetic and tribromoacetic acids. Further, the population-weighted concentrations of chlorodibromoacetic acid and tribromoacetic acid, at 0.28 and 0.21 µg/L, respectively, are lower than the population-weighted concentrations of other haloacetic acids associated with cancer risk, which range from 1.1 to 7.8 µg/L. Thus, we anticipate that the uncertainty around cancer potency of chlorodibromoacetic acid and tribromoacetic acid would not significantly affect the estimated cumulative cancer risk due to the haloacetic acids presented here. 

### 3.3. Calculation of Annual and Lifetime Cancer Risk Based on Epidemiological Studies of Disinfection Byproducts

To assess cancer risk based on human data, we relied on epidemiological studies that found an association between the presence of disinfection byproducts in drinking water (marked by the THM4 levels) and increased risk of bladder cancer. Trihalomethane concentrations correlate with HAA9 concentrations in drinking water (Figure 1), and an assumption is made that trihalomethane concentrations may correlate with levels of other carcinogenic disinfection byproducts in tap water. For this portion of our analysis, we used the THM4 occurrence data for 2014–2017 for 48,363 community water systems in the U.S., serving an estimated 86% of the U.S. population. All estimates for attributable cases were calculated together with upper and lower boundaries based on epidemiologically-derived odds ratios for bladder cancer risk.

The largest number of estimated lifetime bladder cancer cases, approximately 400,000 (Table 7), is calculated for large surface water systems serving communities with more than 0.1 million residents. Combined, these systems serve 40% of the total population served by community water systems in the U.S. As expected, a lower risk is associated with small groundwater systems that serve a smaller proportion of the population and generally have lower disinfection byproduct levels. Converting the estimates of disinfection byproduct-attributable lifetime bladder cancer cases into cancer risk, we estimate that lifetime cancer risk from disinfection byproducts is 3.0 × 10^−3^ (2.1 × 10^−4^–5.7 × 10^−3^) for the 279 million people served by community water systems in the U.S. These estimates are significantly greater than the de minimus risk of one-in-a-million (10^−6^). We completed the same analysis just for the water systems included in the UCMR4 monitoring program and obtained very similar risk estimates of 3.2 × 10^−3^ (2.1 × 10^−4^–6.1 × 10^−3^). This similarity in estimates makes sense given that the UCMR4 program includes all large community water systems in the U.S.

We note that different risk estimates are obtained from calculations based on either annual bladder cancer incidence or lifetime risk of a bladder cancer diagnosis. Analysis based on the annual incidence of bladder cancer results in an annual risk of 2.4 × 10^−5^ due to disinfection byproducts. Multiplying the annual cancer risk estimate by 70, the “statistical length of life” used by U.S. EPA for human health risk assessments, results in a lifetime risk of 1.7 × 10^−3^. This value is lower than an estimated lifetime attributable risk of 3.0 × 10^−3^ calculated from data in Table 7 because the lifetime bladder cancer probability is estimated with the National Cancer Institute DevCan software, which uses a longer lifetime of 95 years and accounts for the higher risk of developing bladder cancer with age.

### 3.4. Calculation of Economic Costs due to Estimated Bladder Cancer Cases Associated with Disinfection Byproducts

We calculated possible costs of medical treatment that would be incurred annually for disinfection byproduct-attributable bladder cancer cases. Bladder cancer is the seventh most common cancer in the U.S. [45]. Around 2.4% of the U.S. population will be diagnosed with bladder cancer during their lifetime, and this cancer occurs three times more frequently in men compared to women. Approximately a quarter of those cases results in death due to cancer, as the 5-year survival rate for bladder cancer is 77%, according to the 2009–2015 data from the National Cancer Institute [45]. Based on annual bladder cancer incidence, we calculate that 6,800 bladder cancer cases every year could be due to disinfection byproducts in drinking water. We estimated a cost of $91,000 per case of bladder cancer (2015 U.S. dollars) using Equation (11) and cancer cost estimates from Mariotto and colleagues [46]. When applied to the national estimate of bladder cancer cases attributable to disinfection byproducts in drinking water, this translates into annual overall medical costs of USD 620 million (2015 dollars). The estimates are conservative in that they account for direct medical costs only, and do not account for lost productivity, indirect medical costs, as well as economic costs and losses for family members and/or caregivers for each patient.

### 3.5. Comparison of Epidemiologically-Based and Toxicologically Based Cancer Risk Estimates

To compare risk estimates using toxicological and epidemiological approaches, we focused all analyses on the 3579 systems in the UCMR4 program. For the toxicological estimates, we calculated the combined cancer risk for THM4 and HAA9 based on lower bound, central, and upper bound estimates of the benchmark dose-derived cancer slope factors. The overall toxicologically-based cancer risk was estimated as 7.0 × 10^−5^ (3.5 × 10^−5^–1.3 × 10^−4^) using default body weight and water intake factors and 2.9 × 10^−4^ (1.7 × 10^−4^–6.2 × 10^−4^) when incorporating age sensitivity factors (Figure 4). The epidemiologically-based assessment of cancer risk in Figure 4 is calculated from lower, central, and upper estimates of bladder cancer risk reported in human studies. 

In a side-by-side comparison of toxicological and epidemiological risk estimates, it is important to acknowledge tumor site non-concordance between animal and human studies. While epidemiological research finds an association of disinfection byproducts in drinking water with bladder cancer, tumors at different sites are observed in laboratory animals (see Figure 3). Using the same exposure data, the central estimate of risk based on human data is five-fold greater than the upper bound estimate of risk calculated from animal data with included age sensitivity factors. This comparative analysis suggests that human epidemiological data must be used to the greatest extent possible to capture risks based on real-world exposures. Risk calculations based on animal bioassays are an essential part of risk assessment and mitigation. As Figure 4 demonstrates, the inclusion of age sensitivity factors brings toxicological risk estimates closer to epidemiological risk estimates, while the default weight and water intake factors produce risk estimates that are smaller than epidemiological estimates by approximately 40-fold. 

## 4. Discussion

Toxicological, epidemiological, and mechanistic studies of disinfection byproducts have provided strong evidence for the carcinogenicity of disinfection byproducts. At the same time, individual disinfection byproducts show differences in both cancer potency and overall toxicity [42,57,59,60]. This differential toxicity is reflected in the diversity of carcinogen classifications for individual disinfection byproduct substances published by the U.S. EPA, National Toxicology Program Report on Carcinogens, and International Agency for Research on Cancer (Table 8). In addition to the chemicals listed and analyzed here, there are numerous other contaminants that form during water disinfection, and they can have greater carcinogenic potency than trihalomethanes and haloacetic acids [61].

Here we present the first analysis of the recently published Unregulated Contaminant Monitoring Rule 4 occurrence data for haloacetic acids. Toxicological assessment indicates haloacetic acids have overall greater cancer potency than trihalomethanes. We found that the group of five regulated haloacetic acids is associated with a smaller number of attributable cancer cases compared to the HAA6Br group, suggesting that in addition to HAA5, levels of other haloacetic acids should be lowered in drinking water in order to protect public health.

Side-by-side comparison of toxicological and epidemiological estimates for cancer risk due to disinfection byproducts in drinking water in the United States represents a unique and novel aspect of our study (Figure 4). While there have been prior studies that looked at toxicological and epidemiological risk estimates [61,69,70], this is the first study to conduct such an analysis on a comprehensive national scale. As our study demonstrates, cumulative risks due to disinfection byproducts of 7 × 10^−5^ to 3 × 10^−4^ according to toxicological calculations (central estimates), or 3 × 10^−3^ according to epidemiological estimates, are significantly greater than one-in-a-million (10^−6^) which is considered as *“de minimus”* acceptable risk in contaminant risk assessments published by the U.S. EPA. In the opinion of the authors of this study, the concept of one-in-a-million risk as a health benchmark remains relevant. However, it is important for future research and policy discussions to address the fact that actual cumulative risks are much greater than this goal of minimal cancer risk.

We calculated that around 6800 annual bladder cancer cases and around 828,000 lifetime bladder cancer cases may be due to disinfection byproducts in drinking water. These estimates are based on the overall incidence of bladder cancer, as reported by the U.S. Centers for Disease Control and Prevention, which includes both invasive and non-invasive bladder cancer cases. Using the 2017 census report of 325 million for the U.S. population and the lifetime risk of bladder cancer of 0.02447 as calculated with the DevCan software, we estimate that about 10% of bladder cancer cases are due to disinfection byproducts (828,000 lifetime bladder cancer cases out of nearly 8 million for the total U.S. population). The U.S. EPA reported that the best estimate for the number of disinfection byproduct-attributable annual bladder cancer cases is between 731 and 6720, depending on the method of calculation [71]. Thus, the number of 6800 annual bladder cancer cases calculated here is in alignment with the earlier analysis.

We recognize the scientific uncertainties associated with the calculation of health risks from toxicology data. For example, cancer slope factors calculated here for dibromoacetic acid, bromochloroacetic acid, and bromodichloroacetic acid used liver tumor data in mice as the most sensitive endpoint across species and tissues. In animal bioassays, exposure-related tumors were observed in different organs (Figure 3), yet not in the bladder, the target site observed in human studies. Translation of animal bioassay data to human risk requires the risk assessors to make certain process decisions and assumptions, such as the use of 5% versus 10% excess risk as the benchmark response level as well as assumptions about the length of exposure, water intake, and age sensitivity factors for early life stages. Even with age sensitivity factors, risk calculations based on animal data might not capture the full range of susceptibility of the fetus, infant, and young child to carcinogens. 

The limitations in epidemiological studies also can influence the reliability of risk estimates. Accurate human exposure assessment generally is challenging. A number of factors could influence epidemiological risks, including confounding risk factors like tobacco smoke exposure, occupational exposures, and co-occurring arsenic contamination [16,72]. Genetic marker research has identified subpopulations particularly susceptible to bladder cancer due to disinfection byproduct exposure [73]. Polymorphisms in metabolizing enzymes involved in the processing of ingested toxic chemicals modify the risk due to disinfection byproduct exposure, although these modifying effects differ in populations with different genetic backgrounds, such as Asian populations versus Caucasian populations, as noted in a recent systematic review [74]. Finally, epidemiological studies focus on exposure to one contaminant or group of contaminants, such as THM4, and the assumption is that the THM4 group concentration reflects the presence of other disinfection byproducts. The reliability of this assumption will need to be assessed in future studies.

The latency period in the development of bladder cancer or any other cancer also represents uncertainty in the calculations of bladder cancer cases attributable to disinfection byproducts. Attributable bladder cancer risk is likely due to prior decades of exposure to disinfection byproducts in drinking water, and changes in water disinfection approaches and disinfection byproduct levels would influence both current and future risk. U.S. EPA regulations in Stage I (1998) and Stage II (2006) Disinfectants and Disinfection Byproduct Rules have decreased overall levels of disinfection byproducts throughout drinking water distribution systems. Thus, risks due to current exposures to disinfection byproducts in United States tap water may be lower than what we calculate from estimates of risks based on earlier epidemiological studies.

Finally, there is uncertainty about trade-offs between cancer risk of disinfection byproducts and avoided risks of death and microbial disease thanks to drinking water disinfection. A comparison of toxicological and microbiological risks is beyond the scope of our study and merits future research. Here, we limit ourselves to noting that a side-by-side analysis of toxicological and epidemiological estimates can provide helpful information for risk assessment and risk mitigation. 

## 5. Conclusions

In closing, this study offers a compelling argument for conducting a cumulative risk assessment for both regulated and unregulated contaminants. Regulated disinfection byproducts constitute just a portion of the contaminants that form during the disinfection process. The inclusion of unregulated haloacetic acids in a toxicologically-based framework increases the likelihood that a cancer risk assessment for disinfection byproducts accurately reflects risk.

Additionally, our analysis highlights the value of using human data in health risk assessments. Epidemiological approaches capture real-world risks from drinking water contaminant mixtures in ways that, at present, cannot be fully assessed by toxicological studies. Thus, we hope that the present study will facilitate the application of epidemiological information in public policy development. 

## Figures and Tables

**Table 1:** Input data for analysis of haloacetic acid occurrence based on the national contaminant occurrence dataset [15].

Disinfection Byproduct	Number of Community Water Systems Included in the Analysis	Population Weighted-Average Concentration (µg/L)	HAA Group	
Monochloroacetic acid	29,672	0.48	HAA5	
Dichloroacetic acid	29,673	7.8	HAA5	
Trichloroacetic acid	29,669	6.4	HAA5	
Monobromoacetic acid	29,669	0.24	HAA5	HAA6Br
Dibromoacetic acid	29,669	1.2	HAA5	HAA6Br
Tribromoacetic acid	986	0.21		HAA6Br
Bromochloroacetic acid	8024	2.9		HAA6Br
Bromodichloroacetic acid	992	1.1		HAA6Br
Chlorodibromoacetic acid	986	0.28		HAA6Br

**Table 2:** Input data for analysis of haloacetic acid occurrence based on the Unregulated Contaminant Monitoring Rule 4 (UCMR4) dataset [15].

Disinfection Byproduct	Number of Community Water Systems Included in the Analysis ^a^	Population Weighted-Average Concentration (µg/L)
**HAA5**	3576	19.1
**HAA6Br**	3579	7.0
**HAA9**	3576	29.4

^a^ Data on five haloacetic acids (HAA5) and nine chlorinated and brominated haloacetic acids (HAA9) was unavailable for three systems in the published UCMR4 dataset.

**Table 3:** Human-equivalent cancer slopes derived from benchmark dose modeling of animal data.

Disinfection Byproduct and Source of Animal Toxicology Data	Benchmark Dose Calculated from Animal Bioassay (mg/kg-day) ^a^	Human-Equivalent Cancer Slope Factor (lower 95% Confidence Limit) ^b^ (mg/kg-day)^−1^	Benchmark Dose Calculated from Animal Bioassay (mg/kg-day) ^a^	Human-Equivalent Cancer Slope Factor (lower 95% Confidence Limit) ^b^ (mg/kg-day)^−1^
*Benchmark response*	*5% excess risk*		*10% excess risk*	
Bromochloroacetic acid [26]	1.0 (0.7–1.6)	0.463	2.0 (1.3–3.2)	0.451
Bromodichloroacetic acid [27]	2.8 (1.6–9.1)	0.185	5.7 (3.2–18.7)	0.180
Dibromoacetic acid [25]	2.2 (1.4–3.8)	0.210	4.5 (3.0–7.7)	0.205

^a^ Benchmark doses modeled for 5% or 10% extra risk of tumor incidence in the liver of mice, the most sensitive site in exposed laboratory animals; central estimates, as well as upper and lower confidence limits, are reported. ^b^ Cancer slope factors were calculated using the lower 95% confidence limit of the benchmark dose and transformed to the human-equivalent using Equation (3).

**Table 4:** Human cancer slope factors and cancer risk concentrations reported in other studies.

Disinfection Byproduct	Cancer Slope Factor Reported by OEHHA ^a^ (mg/kg/day^−1^)	OEHHA One-in-a-Million Cancer Risk Benchmark Concentration (µg/L)	Cancer Slope Factor Reported by the U.S. EPA ^a^ (mg/kg-day^−1^)	U.S. EPA One-in-a-Million Cancer Risk Benchmark Concentration (µg/L)
Dibromoacetic acid	0.250	0.03, 2020 [30]	N/A	N/A
Bromodichloromethane	0.087	0.06, 2018 [42]	0.062	0.6, 1993 [51]
Trichloroacetic acid	0.071	0.1, 2020 [30]	0.067	0.5, 2011 [52]
Dibromochloromethane	0.045	0.1, 2018 [42]	0.084	0.4, 1990 [53]
Dichloroacetic acid	0.041	0.2, 2020 [30]	0.048	0.7, 2003 [54]
Chloroform	0.014	0.4, 2018 [42]	N/A	N/A
Bromoform	0.011	0.5, 2018 [42]	0.008	4.0, 1990 [55]

^a^ Cancer slope factors are based on the lower 95% confidence limit of the benchmark dose or point of departure.

**Table 5:** Benchmark doses and cancer risk concentrations for haloacetic acids and trihalomethanes.

Disinfection Byproduct ^a^	Benchmark Doses from Animal Bioassay, 5% Excess Cancer Risk (mg/kg-day)	Concentrations Corresponding to One-in-a-Million Cancer Risk ^c^ (µg/L)	Source of the Risk Benchmark
Bromochloroacetic acid	1.0 (0.7–1.6) ^b^	0.02	Calculated in this study
Chlorodibromoacetic acid	Not Available	0.02	Applied by read-across from bromochloroacetic acid
Bromodichloroacetic acid	2.8 (1.6–9.1) ^b^	0.04	Calculated in this study
Dibromoacetic acid	2.2 (1.4–3.8) ^b^	0.04	Calculated in this study
Tribromoacetic acid	Not Available	0.04	Applied by read-across from dibromoacetic acid
Trichloroacetic acid	8.1 (4.4–28.8)	0.1	OEHHA 2020 [30]
Dichloroacetic acid	32.7 (7.9–40.2)	0.2	OEHHA 2020 [30]
HAA5 group	Not applicable	0.1	Calculated in this study
HAA6Br group	Not applicable	0.03	Calculated in this study
HAA9 group	Not applicable	0.06	Calculated in this study
Bromodichloromethane	11.1 (3.9–21.9)	0.06	OEHHA 2018 [42]
Dibromochloromethane	26.2 (7.5–39.1)	0.1	OEHHA 2018 [42]
Chloroform	33.4 (14.1–51.6) ^d^	0.4 ^e^	OEHHA 2018 [42]
Bromoform	31.0 (18.7–82.7)	0.5	[42]
THM4 group	Not applicable	0.15	Evans et al. [16]

^a^ There is no evidence of carcinogenicity for monobromoacetic acid and monochloroacetic acid. ^b^ Benchmark doses were modeled using 5% extra risk of tumor incidence in the liver of mice, the most sensitive site in exposed laboratory animals. ^c^ Risk-specific concentrations were calculated based on 95th percentile drinking water intake and adjustment factors to account for early-life sensitivity. ^d^ Benchmark modeling results for chloroform vary depending on the specific bioassay [42,56]; benchmark dose data reported here are from California OEHHA modeling of the data from the National Cancer Institute 1976 report on chloroform carcinogenicity [56]. ^e^ The benchmark, as reported by California OEHHA, is based on the geometric mean of cancer slope factors from five different animal studies [42].

**Table 6:** Toxicological estimates for attributable lifetime cancer cases and cumulative cancer risks due to haloacetic acids and trihalomethanes.

Chemical Group	Cancer Estimates Using Default Parameters ^a,b^	Cancer Estimates Using Age Sensitivity Factors ^a,c^
*Lifetime cancer cases calculated using the national tap water dataset (thousands)*		
THM4 ^d^	3.1 (1.7–10.2)	19.1 (10.2–57.2)
HAA5	4.0 (2.0–8.4)	18.5 (8.6–40.1)
*Lifetime cancer cases calculated for systems in the UCMR4 program (thousands)*		
HAA5	2.9 (1.5–6.1)	13.4 (6.2–29.1)
HAA6Br	8.6 (4.4–12.8)	32.1 (21.4–42.8)
HAA9	10.9 (5.4–17.5)	41.4 (25.3–76.0)
THM4 ^d^ and HAA9	12.8 (6.5–24.1)	53.7 (31.9–112.8)
*Lifetime cancer risk calculated for systems in the UCMR4 program*		
Cumulative cancer risk estimates for THM4 ^d^ and HAA9	7.0 × 10^−5^ (3.5 × 10^−5^–1.3 × 10^−4^)	2.9 × 10^−4^ (1.7 × 10^−4^–6.2 × 10^−4^)

^a^ Estimates are based on the benchmark dose and the corresponding 90% confidence intervals. The upper bound estimate of cancer cases corresponds to the cancer slope factor calculated from the lower bound estimate of the benchmark dose, and vice versa. ^b^ Default parameters use the adult drinking water intake of 2 L/day and body weight of 70 kg. ^c^ Susceptibility factors include an age-sensitivity adjusted 95th percentile drinking water intake of 0.129 L/kg-day for haloacetic acids [30] and 0.18 L/kg-day for trihalomethanes [42]. ^d^ Cancer risk estimates for THM4 are based on benchmark doses reported in Table 5; chloroform benchmark doses are from modeled National Cancer Institute data [42,56].

**Table 7:** Lifetime bladder cancer cases attributable to disinfection byproducts in drinking water (thousands of cases).

Water Source	Population Served	Up to 20 ug/L	20–40 ug/L	40–60 ug/L	60–80 ug/L	Above 80 ug/L	All Concentrations		
Groundwater	10,000 or less	13.2 (0–29.4)	13.7 (0–27.4)	6.7 (1–11.9)	3.2 (1.2–4.9)	2.3 (0.3–5.1)	39.2 (2.5–78.6)	141.5 (10.2–276.5)	828.1 (57.2–1590.2)
	10,001 to 100,000	17.1 (0–37.8)	28.4 (0–56.8)	16.9 (2.7–29.6)	3.6 (1.3–5.6)	0.5 (0.1–1)	66.4 (4–130.8)		
	More than 100,000	4.4 (0–9.7)	14.6 (0–28.5)	11.6 (1.8–20.4)	5.3 (1.8–8.4)	0 (0–0)	35.9 (3.6–67)		
Surface Water	10,000 or less	5.2 (0–11.4)	22.8 (0–44.9)	26.5 (4.2–46.5)	10.6 (3.9–16.4)	5.1 (1–10.6)	70.1 (9.1–129.8)	686.6 (47.1–1313.6)	
	10,001 to 100,000	27.5 (0–60.9)	100.1 (0–197.8)	74.4 (10.9–131.3)	9.7 (3.5–15.2)	4.9 (0.6–11.2)	216.7 (15–416.4)		
	More than 100,000	35.4 (0–78.4)	217.6 (0–427.9)	131.1 (19.2–231.4)	10.8 (3.8–17.1)	4.9 (0–12.6)	399.9 (23–767.4)		

**Table 8:** Halogenated disinfection byproducts classified as carcinogens by authoritative government agencies.

Disinfection Byproduct	International Agency for Research on Cancer (IARC)	U.S. Environmental Protection Agency	National Toxicology Program (U.S.)
*Trihalomethanes*			
Bromodichloromethane	Possibly carcinogenic to humans (Group 2B) [62];	Probable human carcinogen (Group B2) [51]; Likely to be carcinogenic to humans [63];	Reasonably anticipated to be a human carcinogen [64];
Bromoform	Unclassifiable as to carcinogenicity in humans (Group 3) [62];	Probable human carcinogen (Group B2) [55]; Likely to be carcinogenic to humans [63];	
Chloroform	Possibly carcinogenic to humans (Group 2B) [59];	Probable human carcinogen (Group B2) [65]; Likely to be carcinogenic to humans by all routes of exposure under exposure conditions that lead to cytotoxicity and regenerative hyperplasia in susceptible tissues; not likely to be carcinogenic to humans by any route of exposure under exposure conditions that do not cause cytotoxicity and cell regeneration [65];	Reasonably anticipated to be a human carcinogen [66];
Dibromochloromethane	Not classifiable as to its carcinogenicity to humans (Group 3) [67];	Possible human carcinogen (Group C) [53];	
*Haloacetic acids*			
Dichloroacetic Acid	Possibly carcinogenic to humans (Group 2B) [68];	Likely to be carcinogenic to humans [54];	Reasonably anticipated to be a human carcinogen [57]
Dibromoacetic acid	Possibly carcinogenic to humans (Group 2B) [67];		Reasonably anticipated to be a human carcinogen [57]
Bromochloroacetic acid	Possibly carcinogenic to humans (Group 2B) [67];		Reasonably anticipated to be a human carcinogen [57]
Bromodichloroacetic acid			Reasonably anticipated to be a human carcinogen [57]
Trichloroacetic acid	Possibly carcinogenic to humans (Group 2B) [68];	Suggestive evidence of carcinogenic potential [52];	
Chlorodibromoacetic acid			Reasonably anticipated to be a human carcinogen [57]
Tribromoacetic acid			Reasonably anticipated to be a human carcinogen [57]
